# Does the association between white matter hyperintensity severity and Alzheimer’s disease indicate a primary role of cerebrovascular disease in Alzheimer’s disease pathogenesis?

**DOI:** 10.1002/alz.085774

**Published:** 2025-01-09

**Authors:** Adam M. Brickman

**Affiliations:** ^1^ Columbia University, New York, NY USA

## Abstract

White matter hyperintensities (WMH) are areas of increased lucency visualized on T2‐weighted magnetic resonance imaging (MRI), including fluid attenuated inversion recovery (FLAIR) sequences. Over the past 15 years we have been examining WMH in studies of cognitive aging among clinical, community‐based, and populations at genetic risk to understand the role of vascular brain injury in Alzheimer’s disease (AD) onset, symptom progression, and pathogenesis. Our findings suggest that regional WMH, particularly when distributed in posterior areas, increase risk for clinical AD and contribute to the clinical course of the disease, even among genetic population with relatively low rates of vascular risk factor, like adults with Down syndrome and with autosomal dominant genetic mutations for AD. While these observations suggest a role of endogenous cerebrovascular disease as a driver or core feature of AD, there has been considerable discussion about the underlying pathophysiology of WMH. Some authors argue that WMH in the context of AD reflect white matter changes secondary to neurodegeneration (so‐called “Wallerian degeneration”). We have argued against this possibility based on four observations. First, experimental data from animal models of vascular brain injury suggest that hypoperfusion injury gives rise white matter injury and downstream tau pathology and neurodegeneration. Second, the anatomical distribution of WMH in AD more closely aligns with the brain’s perfusion patterns and the vulnerability of whatershed regions to changes in blood supply and small vessel injury than with proximity to cortical neuronal injury. Third, the temporal evolution of WMH in the context of AD suggests a contribution of WMH that precedes expected neurodegenerative changes. Finally, we do not view an observed relationship between WMH and risk factors, such as hypertension, as a requirement to establish a cerebrovascular basis of WMH; rather, in total, findings suggest an “endogenous” cerebrovascular component, perhaps mediated by inflammatory changes, genetics, or blood brain barrier breakdown. Understanding the pathophysiological basis of WMH with respect to underlying vasculature may point to novel directions for disease prevention or intervention.

